# Tentative Identification of Volatile Flavor Compounds in Commercial Budu, a Malaysian Fish Sauce, Using GC-MS

**DOI:** 10.3390/molecules17055062

**Published:** 2012-05-03

**Authors:** Hajaratul Najwa Mohamed, Yaakob Che Man, Shuhaimi Mustafa, Yazid Abdul Manap

**Affiliations:** 1 Halal Products Research Institute, Universiti Putra Malaysia, 43400 UPM Serdang, Selangor, Malaysia; Email: hajaratulnajwa.mohamed@gmail.com (H.N.M.); ycm@putra.upm.edu.my (Y.C.M.); 2 Faculty of Biotechnology and Biomolecular Sciences, Universiti Putra Malaysia, 43400 UPM Serdang, Selangor, Malaysia; 3 Faculty of Food Science and Technology, Universiti Putra Malaysia, 43400 UPM Serdang, Selangor, Malaysia; Email: myazid@food.upm.edu.my

**Keywords:** *Budu*, fish sauce, flavors, aroma, volatile compounds

## Abstract

*Budu* is a famous Malaysian fish sauce, usually used as seasoning and condiment in cooking. *Budu* is produced by mixing fish and salt at certain ratio followed by fermentation for six months in closed tanks. In this study, four commercial brands of *Budu* were analyzed for their chemical properties (pH, salt content and volatile compounds). The pH of *Budu* samples ranged from 4.50–4.92, while the salt (NaCl) content ranged between 11.80% and 22.50% (w/v). For tentative identification of volatile flavor compounds in *Budu*, two GC columns have been used, DB-WAX and HP-5MS. A total of 44 volatile compounds have been detected and 16 were common for both columns. 3-Methyl-1-butanol, 2-methylbutanal, 3-methylbutanal, dimethyl disulfide, 3-(methylthio)-propanal, 3-methylbutanoic acid and benzaldehye have been identified as the aroma-active compounds in *Budu* due to their lower threshold values.

## 1. Introduction

In Southeast and East Asian countries, fish sauce has been used extensively as condiment in cooking. This is due to the deep umami taste of fish sauce. Fish sauce is known by different names according to the country, for example, *bakasang* in Indonesia, *patis* in The Philippines, *yu-lu* in China, and *nampla* in Thailand [1]. *Budu* is a traditional Malaysian fish sauce, normally used as condiment and seasoning. This fish sauce is brown or dark brown in color and is made from a mixture of fish and salt. The types of fish that are commonly used in *Budu* manufacturing are *Stolephorus* spp., *Sardinella* spp. or *Decaterus macrosoma* [2]. The fish and salt mixture is fermented in closed tanks at a temperature of 30–40 °C for several months, usually 6 to 12 [1]. After the fermentation process, palm sugar, tamarind, monosodium glutamate and flavoring compounds are added to the mixture. Then, the mixture is boiled, filtered and packaged [3].

*Budu* is originally from the East Coast states of Malaysia and most of the *Budu* manufacturers are located in Kelantan and Terengganu. Like fish sauces in other countries, *Budu* has a unique taste which has been developed during the fermentation process. Enzymes that are present in fish together with some halotolerant and halophile microorganisms induce the fermentation process that results in hydrolysis of the fish proteins, that then produces free amino acids, peptides and ammonia. *Budu* is a very good source of protein and contains a number of essential amino acids. Since *Budu* sauces have a high salt concentration, the growth of pathogenic bacteria is under controlled and the salt results in the typical “umami” taste and aroma [4].

The analyses of volatile compounds in fish sauce from different countries have been studied since a decade ago by using different methods and sample preparations [5,6,7]. Flavor is a very important characteristic since it is used to measure the quality of a fish sauce. However, no information on volatile flavor compounds of *Budu* produced commercially, has been reported. Therefore, the aim of this study is to identify the volatile compounds that contribute to the special aroma in *Budu* by using Gas Chromatography-Mass Spectrometry (GC-MS). In addition, we also determined the pH and salt content of *Budu*.

## 2. Results and Discussion

### 2.1. pH and Salt Content of Commercial *Budu*

Table 1 shows the pH and salt content data for four *Budu* samples. The pH of the samples ranged from 4.50–4.92. The average pH of fish sauce from various Southeast and East Asia countries such as Burma, China, Japan, Malaysia, Philippines, Vietnam and Thailand was 5.3–6.7 [8].

**Table 1:** pH and NaCl content of four *Budu* samples.

Samples	pH	Salt content (%, w/v)
A	4.77 ± 0.01 ^b^	22.5 ± 0.90 ^a^
B	4.50 ± 0.01 ^c^	11.8 ± 0.80 ^b^
C	4.78 ± 0.00 ^b^	21.9 ± 1.79 ^a^
D	4.92 ± 0.02 ^a^	20.9 ± 0.17 ^a^

* pH and salt content are expressed as means ± S.D, means are obtained from duplicates of three samples. Different lowercase letters (a, b, c) in the same column indicate statistical differences (*p* < 0.05).

Therefore, the result in this study was lower compared to the previous work. Initially, the pH of fish sauce is neutral, however it decreases to acidic during fermentation [9]. In most cases, the acidic pH of fermented foods is correlated with the formation of organic acids during fermentation. Additionally, the types of fish used in *Budu* production also contribute to the different rates of pH decrease. This is because each species has its own buffering capacity of muscle protein [10]. Apart from that, the release of free amino acids from proteins and large polypeptides also contributes to the lowered pH of *Budu*. Rieybroy *et al*. [11] mentioned that pH is a major determinant to assure the safety of *som-fug*, a Thai traditional fermented fish. The sufficiently low pH will avoid spore germination, hence ensuring the shelf stability of a product. 

The salt (NaCl) content of *Budu* samples ranged between 11.80% and 22.50% (w/v) (Table 1). Rosma *et al*. have also reported the salt content of unprocessed *Budu* and their results varied between 21.50% and 25.70% (w/v). Meanwhile, the average NaCl content of fish sauces collected from different Southeast and Asia countries (Burma, Malaysia, Thailand, Philippines, Vietnam, China and Japan) was 26.0% (w/v) [8]. This value was similar to the average NaCl content presented by Mizutani *et al*. Fish sauce samples from 11 Asian countries have been analyzed for salt content and the average value was 25.9 % (w/v) [12]. Consequently we can state that the NaCl content of *Budu* samples that has been obtained in this study was much lower compared to the previous study. The salt content is a very important aspect that should be considered in *Budu* manufacturing since it could influence the quality of the *Budu*. This is because high salt concentration could kils or retard the growth of spoilage microorganisms and pathogenic bacteria during fermentation. As a result, *Budu* samples that contain high salt content will be free from pathogenic microbes contamination such as *Escherichia coli*, *Coliform*, *Vibrio parahaemolyticus* and *Vibrio cholera*. The excessively ionic atmosphere in *Budu* could inactivate ion of the microbial endogenous enzymes and halt the metabolic actions in pathogenic cells [9]. According to the Malaysian Food Act 1983 and Regulation 1985 [13], *Budu* must contain more than 15.0% salt. This is to prevent the growth of spoilage bacteria and maggots that could produce rotten smells and reduce the quality of fish sauce [9,14]. Among four *Budu* samples that have been analyzed, only one samples showed a salt content that lower than 15.0%.

### 2.2. Identification of Flavour Volatile Compounds in *Budu* Samples by Using GCMS

A total of 45 volatile compounds were isolated from *Budu* samples by using two columns, DB-WAX and HP-5MS in which 16 of them were detected by both columns. All of them were identified by matching with NIST08.L MS library of the MSDChem workstation (similarity ratio >50%). Volatile compounds and their odor descriptors of four *Budu* samples are showed in Table 2.

**Table 2:** Volatile compounds and their odor descriptors of four *Budu* samples.

No	Volatile Compounds	Total Area (%) ^a^		Odor Threshold (ppm)	Odor Description	References
		DB-WAX	HP-5MS			
1	Ethanol	85.27	25.53	950,000 ^d^	Alcoholic	[15,16]
2	(*E*)-2-Penten-1-ol	ND ^b^	0.53	89.2 ^d^	Green, plastic	[15]
3	2-Methyl-1-propanol	1.08	ND	6,505.2 ^d^	Solvent like, malty, pungent	[15,16]
4	1-Penten-3-ol	8.53	ND	358.1 ^d^	Burnt, meaty, pungent	[15,17]
5	2-Methyl-1-butanol	4.83	0.41	15.9 ^d^	Fusel oil, butter	[15,16]
6	3-Methyl-1-butanol	3.57	0.71	4 ^d^	Balsamic, burnt, malt	[6,15,18]
7	1-Pentanol	2.77	ND	150.2 ^d^	Green, wax	[15]
8	Propanal	12.16	ND	15.1 ^d^	Pungent	[15,18]
9	2-Methylpropanal	29.58	17.33	1.5 ^d^	Nutty	[15]
10	2-Methylbutanal	27.03	25.21	1 ^d^	Nutty, buttery, oily	[15,19,20]
11	3-Methylbutanal	44.74	50.73	1.1 ^d^	Almond, nutty, buttery	[15,19]
12	Pentanal	8.40	ND ^b^	0.012–0.042 ^f^	Almond, malt, pungent	[17]
13	Hexanal	6.32	0.32	5 ^d^	Fishy, Grassy	[15,21,22]
14	Heptanal	ND	0.12	2.8 ^d^	Fishy, oily, fatty, sweet, nutty	[15,17,23,24]
15	Acetone	3.09	ND	100	Light ethereal, nauseating	[25]
16	2-Butanone	4.32	ND	35,400.2 ^d^	Chemical, burnt	[26]
17	1-(1*H*-Pyrrol-2-yl)-ethanone	ND	0.06	NE ^c^	Musty, nutty, caramel	[27]
18	1-Hydroxy-2-propanone	0.79	ND	1,800 ^d^		
19	Furan	0.89	ND	NE	NE	
20	2-Ethylfuran	17.76	7.07	2.3 ^d^	Rubber, pungent, burnt	[15,17]
21	2-Pentylfuran	0.46	0.05	5.8 ^d^	Beany, grassy, licorice	[15,17,28]
22	*cis*-2-(2-Pentenyl)furan	ND	0.24	NE	Beany, grassy, buttery	[29]
23	*trans*-2-(2-Pentenyl)furan	1.21	ND	NE	Beany, grassy, buttery	[29]
24	2-Furanmethanol	5.38	ND	4,500.5 ^d^	Oily, burnt sugar	[30]
25	Dimethyl disulfide	ND	1.65	1.1^ d^	Cooked cabbage, vegetable, onion, putrid	[15,17,31]
26	3-(Methylthio)-propanal	ND	0.88	0.45 ^d^	Meaty, potato	[15]
27	Trimethylamine	ND	2.72	0.000037 ^f^	Fishy	[32]
28	2,6-Dimethylpyrazine	0.49	0.07	0.2–9 ^f^	Roasted, coffee, peanut	[15,28]
29	Benzaldehyde	1.59	0.38	750.89 ^d^	Bitter almond, burnt sugar	[15,17,30]
30	Benzeneacetaldehyde	2.27	1.75	NE	NE	
31	1-Isocyano-4-methyl-benzene	ND	0.06	NE	NE	
32	Benzoic acid	ND	1.74	NE	Odorless	
33	Acetic acid	55.61	127.38	24 ^e^	Pungent, vinegar like	[22,30,33]
34	Butanoic acid	1.43	ND	0.24 ^f^	Rancid, buttery, acidic, sour, cheesy	[30,34]
35	2-Methyl-propionic acid	2.14	ND	6,550.5 ^d^	Cheesy, butter	[15,30]
36	3-Methyl-butanoic acid	15.84	ND	0.12–0.7 ^f^	Rancid, sweaty odor, cheesy	[33,35]
37	Hexadecane	ND	11.07	NE	Odorless	
38	Pentadecane	34.87	13.31	NE	Odorless	
39	8-Heptadecane	ND	0.21	NE	Odorless	
40	Tetradecane	ND	0.69	NE	Odorless	
41	2,6,10,14-Tetramethyl-hexadecane	ND	0.30	NE	Odorless	
42	2,6,11,15-Tetramethyl-hexadecane	ND	0.28	NE	Odorless	
43	2,6,11-Trimethyl-dodecane	ND	0.35	NE	Odorless	
44	Methylene chloride	0.31	ND	214 ^e^	NE	
45	Trichloromethane	0.83	10.54	3.80 ^e^	NE	

^a^ Total area (%) of volatile compound for four *Budu* samples; ^b^ not detectable; ^c^ not estimated; ^d^ odor threshold obtained from [15]; ^e^ odor threshold obtained from [36]; ^f^ odor threshold obtained from [37].

#### 2.2.1. DB-WAX

**Figure 1:**
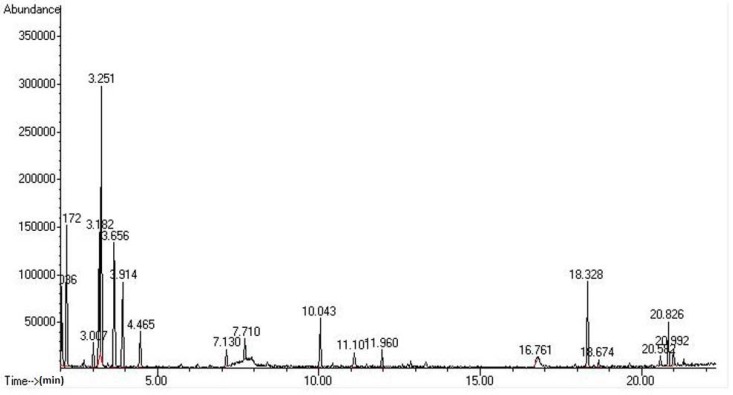
GC profile of volatile compounds extracted from *Budu* 1 using a DB-WAX column.

Figure 1 shows the GC profile of volatile components in *Budu* 1 while Table 3 shows the retention time and percentage of area of the four *Budu* samples by using a DB-WAX column. A total of 30 volatile compounds were detected, consisting of nine classes, including six alcohols, six aldehydes, three ketones, five furans, one nitrogen containing compound, two aromatic compounds, four acids, one hydrocarbon and two halogenated compounds.

**Table 3:** Retention time and percentage of area of volatile compounds detected in four *Budu* samples by using DB-WAX column.

Peak No.	Volatile compounds	Budu 1		Budu 2		Budu 3		Budu 4	
		RT ^1^	Area (%) ^2^	RT ^1^	Area (%) ^2^	RT ^1^	Area (%) ^2^	RT ^1^	Area (%) ^2^
	*Alcohols*								
9	Ethanol	3.658	14.52 ± 3.71 ^a^	3.657	31.56 ± 2.12 ^b^	3.655	9.03 ± 0.82 ^c^	3.656	30.12 ± 1.36 ^b,d^
14	2-Methyl-1-propanol	ND ^3^	ND ^3^	ND ^3^	ND ^3^	ND ^3^	ND ^3^	8.381	1.08 ± 0.02
15	1-Penten-3-ol	10.038	3.41 ± 0.15 ^a^	10.048	1.39 ± 0.15 ^b^	10.038	3.73 ± 0.21 ^c^	ND	ND
16	2-Methyl-1-butanol	ND	ND	ND	ND	ND	ND	11.077	4.83 ± 0.73
17	3-Methyl-1-butanol	11.198	1.82 ± 0.10 ^a^	11.112	1.75 ± 0.13 ^a^	ND	ND	ND	ND
19	1-Pentanol	11.960	0.95 ± 0.02 ^a^	11.965	0.43 ± 0.01 ^b^	11.960	0.61 ± 0.01 ^c^	11.960	0.78 ± 0.05 ^d^
	*Aldehydes*								
1	Propanal	2.036	4.10 ± 0.69 ^a^	2.036	3.23 ± 0.59 ^b^	2.036	4.10 ± 0.64 ^a^	2.033	0.73 ± 0.26 ^c^
3	2-Methylpropanal	2.172	10.46 ± 2.1 ^a^	ND	ND	3.000	10.02 ± 0.70 ^a^	3.004	9.10 ± 0.33 ^a^
6	2-Methylbutanal	3.185	8.73 ± 1.96 ^a^	3.187	2.53 ± 0.29 ^b^	3.182	6.51 ± 0.51 ^c^	3.182	9.26 ± 0.23 ^a^
7	3-Methylbutanal	3.252	18.59 ± 3.34 ^a^	4.401	6.11 ± 0.76 ^b^	4.368	8.91 ± 0.96 ^c^	4.367	11.13 ± 1.04 ^a,c^
11	Pentanal	4.462	3.42 ± 0.01 ^a^	4.466	1.81 ± 0.23 ^b^	4.461	1.69 ± 0.03 ^c^	4.466	1.48 ± 0.06 ^d^
13	Hexanal	7.708	2.05 ± 0.33 ^a^	7.710	1.74 ± 0.22 ^a^	7.710	2.53 ± 0.19 ^b^	ND	ND
	*Ketones*								
4	Acetone	ND	ND	2.187	3.09 ± 0.25	ND	ND	ND	ND
5	2-Butanone	3.007	1.57 ± 0.28 ^a^	ND	ND	3.005	1.67 ± 0.06 ^a^	3.005	1.08 ± 0.10 ^b^
21	1-Hydroxy-2-propanone	ND	ND	ND	ND	ND	ND	12.755	0.79 ± 0.05
	*Furans*								
2	Furan	ND	ND	ND	ND	ND	ND	2.070	0.89 ± 0.20
10	2-Ethylfuran	3.914	6.15 ± 1.46 ^a^	3.914	6.30 ± 1.22 ^a^	3.914	4.44 ± 0.62 ^b^	3.914	0.87 ± 0.33 ^c^
18	2-Pentylfuran	ND	ND	11.460	0.46 ± 0.11	ND	ND	ND	ND
20	*trans*-2-(2-Pentenyl)furan	ND	ND	12.800	1.21 ± 0.45	ND	ND	ND	ND
30	2-Furanmethanol	20.826	2.87 ± 0.16 ^a^	20.826	0.89 ± 0.10 ^b^	ND	ND	20.831	1.62 ± 0.15 ^c^
	*Nitrogen containing compound*								
22	2,6-Dimethylpyrazine	ND	ND	ND	ND	ND	ND	13.460	0.49 ± 0.03
	*Aromatic compounds*								
25	Benzaldehyde	18.676	0.65 ± 0.09 ^a^	ND	ND	18.672	0.94 ± 0.06 ^b^	ND	ND
29	Benzeneacetaldehyde	20.582	0.87 ± 0.07 ^a^	ND	ND	20.585	0.84 ± 0.05 ^a^	20.582	0.56 ± 0.02 ^b^
	*Acids*								
23	Acetic acid	16.753	5.04 ± 0.82 ^a^	16.679	19.23 ± 0.84 ^b^	16.679	25.18 ± 0.97 ^c^	16.669	6.16 ± 0.20 ^a^
26	Butanoic acid	ND	ND	20.479	1.43 ± 0.03	ND	ND	ND	ND
27	2-Methylpropionic acid	ND	ND	ND	ND	19.630	1.20 ± 0.08 ^a^	19.630	0.94 ± 0.06 ^a^
31	3-Methylbutanoic acid	20.982	1.53 ± 0.15 ^a^	20.982	3.63 ± 0.48 ^b^	20.987	4.16 ± 0.45 ^b^	20.982	6.52 ± 0.23 ^c^
	*Hydrocarbons*								
24	Pentadecane	18.332	6.84 ± 0.36 ^a^	18.333	10.19 ± 0.68 ^b^	18.333	9.24 ± 0.54 ^c^	18.333	8.60 ± 0.25 ^c^
	*Chlorinated compounds*								
8	Methylene chloride	ND	ND	ND	ND	ND	ND	3.457	0.31 ± 0.04
12	Trichloromethane	ND	ND	ND	ND	ND	ND	5.724	0.83 ± 0.05

^1^ Retention time; ^2^ Area of each compounds are expressed as means ± S.D; means are obtained from duplicates of three samples. Different letters (a, b, c, d) in the area of each compound shows statistical differences (*p* < 0.05); ^3^ Not detectable.

There are three main contributing odor features in fish sauce, consisting of cheesy, meaty and ammonical notes [38]. These notes are contributed by different classes of volatile compounds. In the alcohol group, ethanol was the most important compound in all *Budu* samples since its concentration was higher. Ethanol is produced during fermentation when sugar is converted into ethanol due to the action of microorganisms. However, according to Girand and Durance [39], fatty acids in fish may yield alcohol through secondary decomposition of hyperoxides. Among the four *Budu* samples, *Budu* 4 showed the highest ethanol level. Even though the content of ethanol was high, it may not contribute to the flavor of fish sauce since its threshold value was high. In contrast, the long straight chain alcohol, for example, 1-pentanol (green, wax odor) may provide aroma to the *Budu* due to the low threshold value. Besides that, branched chain alcohols such as 2-methyl-1-propanol (solvent like), 1-penten-3-ol (burnt, meaty, pungent), 2-methyl-1-butanol (fusel oil, butter) and 3-methyl-1-butanol (balsamic, burnt, malt) also confer significant flavor to *Budu* [6,15,16,17,18]. According to Table 3, the four *Budu* samples contain very low concentrations of these alcohols or they were not detected at all.

Aldehydes are very important compounds that could give significant aromas, either pleasant or rancid, to food stuffs due to their low threshold values [40]. During fermentation, aldehydes are formed through lipid oxidation. However, some aldehydes, namely branched-short chain aldehydes, are generated from the deamination of amino acids [41]. Some aldehydes have been identified in *Budu* samples and they consisted of straight, branched-chain and aromatic aldehydes.

The most prominent aldehyde in the four samples was 3-methylbutanal of which the highest content was found in *Budu* 1 (18.59%), then *Budu* 4 (11.13%), followed by *Budu* 3 (8.91%) and the lowest content was in *Budu* 2 (6.11%). Besides 3-methylbutanal, other aldehydes that showed high amount in the samples were 2-methylpropanal and 2-methylbutanal. This was probably due to high fat content in the raw fish used [15]. Straight and branched chain such as propanal, pentanal, hexanal, 2-methylpropanal, 2-methylbutanal and 3-methylbutanal generally give fishy and grassy, nutty and pungent scents [15,16,17,18,19,20,21,22,23,24]. Based on study done by Giri *et al*. [15], each of these compounds possesses their own specific odors. For instance, propanal gives a pungent smell, hexanal provides a fishy, grassy aroma, 2-methylpropanal, 2-methylbutanal and 3-methylbutanal produce nutty odors. Therefore, all these compounds play a key role in the meaty notes of *Budu* [7]. Overall, the amount of aldehydes in the four *Budu* samples was quite high, even though some of them were not detected in *Budu* 2 and *Budu* 4.

Only three ketone compounds were detected in the *Budu* samples and not all of them were present in each sample. Peralta *et al*. [42] have observed that many ketones may contribute to the cheesy odor of fish sauce, although in another study, they reported that ketones had less impact on the flavor of *Budu* because of their high threshold values [7].

Based on studies that have been reported before, furans have a great impact in enhancing the aroma of fermented food. Usually, furans are formed through *Amadori* rearrangement pathways or are present in dehydrated or fermented carbohydrate condensates [43]. Oxidation of fatty acids also could produce furans and their derivatives as well [44]. In different *Budu* samples, a total of five furans were identified, but none of the samples contained all the furan compounds. Amongst all the furans, 2-ethyl-furan may give highest impact on the aroma of *Budu* due to its lower threshold value. This compound generates rubber and pungent smells in *Budu* and all samples contained this compound [15,17]. Besides 2-ethylfuran, 2-pentylfuran also had low threshold values. Taylor and Mottram [44] reported that 2-pentylfuran may produce beany, grassy and licorice-like taste in soybean oil. In addition, *trans*-2-(2-pentenyl)furan which diaplays a low odor detection limit also generated the same odor as 2-pentyl-furan [29]. According to Table 2, 2-pentylfuran and *trans*-2-(2-pentenyl)furan were only detected in *Budu* 2. Therefore *Budu* 2 had a characteristic green bean-like aroma which made it different from the other brands of *Budu*. 

2,6-Dimethylpyrazine was the single detected nitrogenous compound, which existed in low levels in *Budu* 4. Meanwhile in the other samples, no nitrogen-containing compounds were identified. Normally, these compounds are produced from Maillard reactions [45] which occur during fermentation. Although present in small amounts, nitrogen-containing heterocyclic pyrazines such as 2,6-dimethylpyrazine could impart roasted, coffee and peanut aromas in several fermented foods [15,28]. For that reason, *Budu* 4 might taste different from other brands with a roasted coffee and nutty flavour. Like aldehydes, nitrogen-containing compounds may be accountable for the meaty notes in *Budu* [7].

Benzaldehyde and benzeneacetaldehyde were two of the aromatic compounds that have been identified. Both *Budu* 1 and *Budu* 3 contained these two compounds, while *Budu* 4 only showed the presence of benzeneacetaldehyde. As for *Budu* 2, both compounds were absent. Pham *et al*. have reported that benzaldehyde produced almond, burnt sugar and sweet odors in the finish products and it was one of the most important compounds that contributes to the aroma of fish sauce. Meanwhile, the effect of the odor of benzeneacetaldehyde was not well understood since this compound has not been reported in other studies of volatile compounds in fish sauce. 

In the group of four volatile acids that have been identified, acetic acid was the most abundant one found in all samples. Acetic acid contributes vinegar-like and pungent aromas to fish sauce [20,30,33]. Although the threshold value of acetic acid is quite high, it might have a significant contribution to the aroma due to its abundance in *Budu*. 3-Methylbutanoic acid, which has a 30 times lower threshold value than acetic acid would impart the rancid, sweaty odor in *Budu* [33,35]. The threshold value for butanoic acid and 3-methylbutanoic acid were 3.89 and 2.45 ppb in the vapour phase, respectively. Due to their low odor detection threshold, Shimoda *et al*. [7] reported that butanoic acid, 3-methylbutanoic and 2-methylpropanoic acids play a major role in contributing cheesy and stinging notes in fish sauce. 

Another volatile compound that was found in high amounts in all *Budu* samples was pentadecane. Decarboxylation of higher non-volatile fatty acids could result in the formation of this long chain hydrocarbon. Although present in high amounts, hydrocarbon compounds, either aliphatic or alicyclic would not give so much effect in flavoring the *Budu*. This is because they are not odiferous and had high threshold values [46,47].

Finally, chlorinated components have also been discovered in the *Budu* samples. Only *Budu* 4 contained these compounds which consisted of methylene chloride and trichloromethane. The existence of these compounds may be associated with contamination of the raw fish used. Previously, Reinert *et al*. [48] have detected several chlorinated derivatives in fish tissue. The same result was obtained by other authors who studied fish contamination [49,50]. The fish probably was contaminated by methylene chloride during exposure to ambient air since the typical sources of this compound were spray painting and other aerosols used [51]. However, according to Alexander *et al*. methylene chloride was less toxic compared to other chlorinated compounds. It also did not affect the odor of *Budu* in view of the fact that it has a high odor threshold [52]. As for trichloromethane which also known as chloroform, its content was also low. Trichloromethane was produced from the reaction of chlorine with organic chemicals in both waste water and raw water. Therefore, the major source of trichloromethane in fish was thought to be sea pollution [53].

#### 2.2.2. HP-5MS

**Figure 2:**
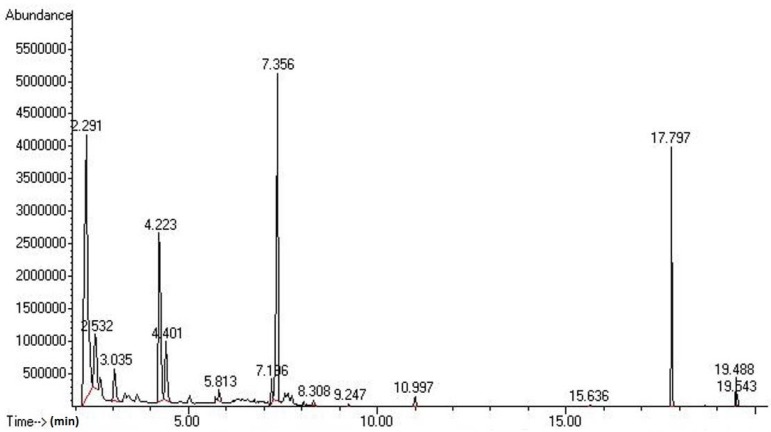
GC profile of volatile compounds extracted from the *Budu* 1 using an HP-5MS column.

The GC profile of the volatile flavor compounds contained in a *Budu* sample using an HP-5MS column is shown in Figure 2. A total of 30 volatile compounds were detected, consisting of nine classes, including six alcohols, six aldehydes, three ketones, five furans, one nitrogen-containing compound, two aromatic compounds, four acids, one hydrocarbon and two halogens. Table 4 lists the retention times and area percentages of the four *Budu* samples.

The number of alcohol compounds that have been identified using the HP-5MS column were less than by using DB-WAX column. All the compounds detected by HP-5MS column were also identified by DB-WAX column except (E)-2-penten-1-ol. This compound has been observed only in *Budu* 2 and it had green, plastic odor [15]. However, since its odor threshold perception was quite high, thus the impact on *Budu* aroma was less. Based on Table 3, 3-methylbutanol was not detected in *Budu* 3 and *Budu* 4, but in Table 4, only *Budu* 3 did not contain this compound. This is due to column separation in which DB-WAX might be better in alcohol separation since the amount of alcohol components being detected by using DB-WAX was greater. 

Five aldehydes have been detected and four of them were also have been identified by the DB-WAX column. By using the HP-5MS column, propanal and hexanal were unable to be detected in the samples with the DB-WAX one did, however, a low level of heptanal has been observed. Together with other straight chain aldehydes, heptanal was responsible for the fishy and nutty aroma in *Budu* [15,17]. A single ketone, 1-(1*H*-pyrrol-2-yl)-ethanone was only detected in *Budu* 4. This compound is a flavor and fragrance agent with musty, nutty-like aroma [27]. Therefore, as mentioned before, *Budu* 4 might possess high level of nutty odor since it contains more compounds that could contribute to that aroma.

Three furan compounds were identified using the HP-5MS column and two of them were already detected using the DB-WAX column. *cis*-2-(2-Pentenyl)furan was the single compound that could not be separated by the DB-WAX column. It has been detected in *Budu* 2 by using the HP-5MS column. However, three furan compounds that were detected by using DB-WAX column could not be separated by the HP-5MS column. They were furan, trans-2-(2-pentenyl)furan and 2-furanmethanol. *cis*-2-(2-Pentenyl)furan reflects the same odour properties as *trans*-2-(2-pentenyl)furan but with lower sensory threshold value [29]. As a result, *cis*-2-(2-pentenyl)furan, which produced beany, grassy and buttery aromas, may have much more impact on *Budu* aroma. 

One of the advantages of using the HP-5MS column was it could result in detection of sulphur containing compounds. In this case, dimethyl disulfide and 3-(methylthio)-propanal were identified in *Budu* samples. The presence of these compounds in fish sauce has been reported previously by some authors [15,17,31]. Sulphur containing compounds like dimethyl disulfide normally originate from the raw fish or form during the fermentation process [54]. Dimethyl disulfide was absent in *Budu* 4 but present in the other three *Budu* samples, while 3-(methylthio)-propanal was observed in all *Budu* samples.

3-(Methylthio)propanal is a sulphur compound and it probably developed from amino acids, in this case, methionine [39]. Both of these compounds have low threshold values meaning that they can be considered as important odor constituents in *Budu* [55]. Dimethyl disulfide gives a cooked cabbage, vegetable, putrid odor [15,17,31] while 3-(methylthio)-propanal contributes to meaty and cooked potato aroma [15]. 

Many nitrogen-containing compounds contribute to the ammonical note of fish sauce. One of them was trimethylamine, which was found in *Budu* 1, *Budu* 2 and *Budu* 4. Trimethylamine is derived from nonbacterial sources, usually raw fish in which antibiotic materials are present [56,57]. This compound has a low sensory threshold and is usually related to fishy note in fish sauce [32]. Besides contributing aroma to the product, trimethylamine also has special characteristics as a spoilage indicator and its concentration has been used to measure the freshness of fish [58]. Besides trimethylamine, 2,6-dimethylpyrazine has also been detected in a sample (*Budu* 4) by using the HP-5MS column. There was in addition aromatic compound, where 1-isocyano-4-methylbenzene has been identified in the sample, besides benzaldehye and benzeneacetaldehyde using the HP-5MS column. However, the amount of this constituent was very low and it was only detected in *Budu* 1, thus it might not be so important as an aroma active component.

**Table 4:** Retention time and percentage of area of volatile compounds detected in four *Budu* samples by using HP-5MS column.

Peak No.	Volatile Compounds	Budu 1		Budu 2		Budu 3		Budu 4	
		RT ^1^	Area (%) ^2^	RT ^1^	Area (%) ^2^	RT ^1^	Area (%) ^2^	RT ^1^	Area (%) ^2^
	*Alcohols*								
1	Ethanol	2.510	7.93 ± 0.16 ^a^	2.530	5.78 ± 0.09 ^b^	2.491	6.89 ± 0.19 ^c^	2.4923	4.93 ± 0.15 ^d^
6	(*E*)-2-penten-1-ol	ND ^3^	ND	4.767	0.53 ± 0.03	ND	ND	ND	ND
8	3-methyl-1-Butanol	5.717	0.28 ± 0.01 ^a^	5.719	0.27 ± 0.01 ^a^	ND	ND	5.706	0.16 ± 0.01 ^b^
9	2-methyl-1-butanol	ND	ND	ND	ND	ND	ND	5.750	0.41 ± 0.02
	*Aldehydes*								
2	2-methyl-propanal	3.016	3.42 ± 0.16 ^a^	3.044	0.74 ± 0.11 ^b^	3.000	2.42 ± 0.09 ^c^	3.004	10.75 ± 0.14 ^d^
4	3-methyl-Butanal	4.205	13.48 ± 0.30 ^a^	4.250	4.37 ± 0.06 ^b^	4.195	4.41 ± 0.10 ^b^	4.197	28.47 ± 0.51 ^c^
5	2-methyl-Butanal	4.379	5.77 ± 0.20 ^a^	4.401	1.53 ± 0.03 ^b^	4.368	3.59 ± 0.28 ^c^	4.367	14.32 ± 0.12 ^d^
11	Hexanal	ND	ND	6.677	0.32 ± 0.02	ND	ND	ND	ND
14	Heptanal	ND	ND	8.212	0.05 ± 0.00 ^a^	8.225	0.05 ± 0.01 ^a^	8.226	0.02 ± 0.01 ^b^
	*Ketones*								
21	1-(1*H*-pyrrol-2-yl)-Ethanone**	ND	ND	ND	ND	ND	ND	11.598	0.06 ± 0.00
	*Furans*								
7	2-ethyl-Furan	5.020	0.97 ± 0.09 ^a^	5.032	4.72 ± 0.29 ^b^	5.004	0.66 ± 0.09 ^c^	5.015	0.72 ± 0.01 ^a,c^
18	2-pentyl-Furan	ND	ND	9.747	0.05 ± 0.00	ND	ND	ND	ND
19	*Cis*-2-(2-Pentenyl)furan**	ND	ND	9.943	0.24 ± 0.01	ND	ND	ND	ND
	*Sulphur containing compounds*								
21	Dimethyl disulfide	5.819	0.86 ± 0.02 ^a^	5.809	0.28 ± 0.01 ^b^	5.7943	0.51 ± 0.01 ^c^	ND	ND
17	3-(methylthio)-Propanal	8.308	0.28 ± 0.01 ^a^	8.302	0.12 ± 0.02 ^b^	8.312	0.14 ± 0.01 ^c^	8.304	0.34 ± 0.01 ^d^
	*Nitrogen containing compounds*								
12	Trimethylamine	6.783	0.86 ± 0.01 ^a^	ND	ND	6.206	1.04 ± 0.09 ^a^	6.524	0.82 ± 0.09 ^a^
16	2,6-Dimethyl-Pyrazine	ND	ND	ND	ND	ND	ND	8.485	0.07 ± 0.01
	*Aromatic compounds*								
17	Benzaldehyde	9.250	0.11 ± 0.01 ^a^	9.240	0.06 ± 0.00 ^b^	9.251	0.14 ± 0.01 ^c^	9.147	0.07 ± 0.01 ^b^
20	Benzeneacetaldehyde	10.804	0.56 ± 0.01 ^a^	11.006	0.21 ± 0.00 ^b^	11.000	0.50 ± 0.02 ^c^	10.998	0.48 ± 0.01 ^c^
23	1-isocyano-4-methyl-Benzene	15.635	0.06 ± 0.01	ND	ND	ND	ND	ND	ND
	*Acids*								
13	Acetic acid	7.005	19.29 ± 1.32 ^a^	7.563	61.57 ± 1.10 ^b^	6.629	35.43 ± 1.08 ^c^	6.794	11.09 ± 0.87 ^d^
22	Benzoic acid	ND	ND	13.649	0.73 ± 0.07 ^a^	13.568	1.01 ± 0.19 ^a^	ND	ND
	Hydrocarbons								
24	Hexadecane	17.797	7.19 ± 0.06^a^	ND	ND	ND	ND	17.797	3.88 ± 0.03 ^b^
25	Pentadecane	ND	ND	17.818	7.34 ± 0.49 ^a^	17.797	5.97 ± 0.01 ^a^	ND	ND
26	8-Heptadecane	ND	ND	19.343	0.07 ± 0.01	ND	ND	ND	0.14 ± 0.01 ^a^
27	Tetradecane	19.491	0.69 ± 0.01	ND	ND	ND	ND	ND	ND
28	2,6,10,14-tetramethyl-Hexadecane	19.543	0.30 ± 0.01	ND	ND	ND	ND	ND	ND
29	2,6,11,15-tetramethyl-Hexadecane	ND	ND	19.543	0.28 ± 0.01	ND	ND	ND	ND
30	2,6,11-trimethyl-Dodecane	ND	ND	ND	ND	19.543	0.21 ± 0.01 ^a^	19.543	0.14 ± 0.01 ^a^
	*Halogen*								
3	Trichloromethane	3.597	3.81 ± 0.96 ^a^	3.647	2.33 ± 0.29 ^a^	3.599	2.74 ± 0.07 ^a^	3.609	1.66 ± 0.79 ^a^

^1^ Retention time; ^2^ Area of each compounds are expressed as means ± S.D; means are obtained from duplicates of three samples. Different letters (a, b, c, d) in the area of each compound shows statistical differences (*p* < 0.05); ^3^ Not detectable.

Only two acids were observed in the samples using the HP-5MS column compared to DB-WAX column which could identify four acids. Acetic acid was the only common acid that has been detected by using both columns. In this experiment, another acid observed in *Budu* samples was benzoic acid. Benzoic acid is odorless but it is commonly used as food preservative. This is due to the ability of benzoic acid to prevent the growth of yeast in food [59]. This compound was found in *Budu* 2 and *Budu* 3. Therefore, the manufacturers of these two brands probably added benzoic acid into their products in order to enhance the shelf life. Benzoic acid could be harmful to human health if its amount in food is in excess of the permitted safety limit. The acceptable amount of benzoic acid in foods is in the range of 0.05 to 0.1 percent by volume [60]. Based on Table 4, the amounts of benzoic acid present in *Budu* 2 and *Budu* 3 were low. Nevertheless, the volatile compounds in this study were not determined quantitatively, hence the amounts of compounds shown in the tables were not the actual amounts that were present in the samples. Therefore, it is not guaranteed that the amount of benzoic acid contained in *Budu* samples is lower or higher than permitted level.

Seven aliphatic hydrocarbons have been separated by HP-5MS column and most of them showed low area percentages. Since hydrocarbons are odorless and have high threshold values, they become insignificant as aroma contributors in *Budu*. In this case, chlorinated compounds have also been found in the samples. However, surprisingly, trichloromethane was present in all the samples, not only in *Budu* 4 as shown in Table 3.

## 3. Experimental

### 3.1. Materials and Chemicals

Four commercial brands of *Budu* were randomly purchased from the local market in Malaysia. All the samples were collected on the same day and kept in the chiller (6 °C) until needed for the pH, salt and volatile compounds analyses. Concentrated nitric acid (HNO_3_) and potassium thiocyanate (KSCN) were obtained from Merck (Darmstadt, Germany). Ammonium iron (III) sulfate dodecahydrate (NH_4_Fe(SO_4_)_2_.12H_2_O) was obtained from Sigma-Aldrich (St. Louis, MO, USA) and silver nitrate (AgNO_3_) was obtained from Fluka (Buchs, Switzerland).

### 3.2. Determination of pH and Salt Content

The pH of *Budu* samples were measured using a pH meter from Mettler Toledo (Columbus, OH, USA). Meanwhile, salt content was determined using the AOAC Official Methods procedure (AOAC, 2000). Briefly, sample (1 mL) was mixed with 0.1 M AgNO_3_ (50 mL) and HNO_3_ (20 mL). The solution was boiled gently by using a hot plate for 15–25 min until all solids except AgCl were dissolved. The solution was cooled using running water. Then, distilled water (50 mL) was added, followed by ferric ammonium sulfate (5 mL) as indicator. The solution was titrated with standardized 0.1 M KSCN solution until the solution turned to permanent brownish-red. The experiment has been done in triplicates and the average volume of KSCN used was obtained. Lastly, the salt content was calculated and expressed as % NaCl. 

### 3.3. Tentative Identification of Volatile Compounds

Three milliliter of each *Budu* sample was introduced into a twenty milliliter headspace vial and was thus ready to be analyzed. Volatile compounds in *Budu* samples were identified using an Agilent-Technologies 7890A GC system equipped with an Agilent-Technologies 5975C Inert MSD with triple-axis detector and Agilent-Technologies G1888 Network headspace sampler (Agilent-Technologies, Little Falls, CA, USA). GC/MS analysis of volatile compounds was performed twice using two different capillary columns, a DB-WAX (30 m × 0.25 mm × 0.25 μm) and an HP-5MS (30 m × 0.25 mm × 0.25 μm) (J&W Scientific, Folsom, CA, USA). The volatile compounds were identified by comparing and matching mass spectra fragment with the NIST08. L MS library. Each sample was run in triplicate.

### 3.4. GC and Headspace Conditions (DB-WAX Column)

The electron ionization mode was used for MS detection with a mass range of *m/z* 29–450. GC was performed in split mode with the split ratio of 10:1. Oven temperature was set at 37 °C for 6 min. Then it was heated up to 100 °C at 10 °C/min for 5 min. Next, it was ramped to 200 °C at 15 °C/min and held for 10 min. From 200 °C, the oven temperature was increased to 230 °C at a programmed rate of 20 °C/min and held for 2 min. The GC injector line temperature was 250 °C and MS transfer line temperature was 230 °C. Helium was used as carrier gas at a flow rate of 1 mL/min. The run time was 35.8 min. Meanwhile, for headspace conditions, the oven, loop and transfer line temperatures were programmed at 80 °C, 90 °C and 100 °C, respectively. The timing for vial equilibration, pressurization, loop fill, loop equilibrium and inject were fixed for 15.0, 0.20, 0.20, 0.05 and 1.00 min, respectively. The temperature of MS quad and MS source were set at 230 °C and 150 °C, respectively.

### 3.5. GC and Headspace Conditions (HP-5MS Column)

Electron ionization mode was used and with the mass range seat at *m/z* 40–400. GC was programmed to split mode with split ratio of 10:1. The oven temperature was kept at 35 °C for 4 min, and then heated from 35 to 100 °C for 4 min at a rate of 20 °C/min. Finally, the temperature was increased to 300 °C at a programmed rate of 15 °C/min for 10 min. The temperature of the GC injector line was 250 °C and temperature of the MS transfer line was 230 °C. The carrier gas was helium with the flow rate of 0.5 mL/min. The run time was 34.58 min. The oven, loop and transfer line temperature for headspace were fixed to 110 °C, 120 °C and 130 °C respectively. Meanwhile, the vial equilibration, pressurization, loop fill, loop equilibrium and inject timing were fixed to 20.0, 0.20, 0.20, 0.05 and 1.00 min respectively The MS source and MS squad temperature were programmed at 230 °C and 150 °C, respectively.

## 4. Conclusions

The pH and salt content of commercial *Budu* were found to be different among brands in this study. In GC-MS analyses, many significant volatile compounds that are responsible for contributing aroma in *Budu* were identified by using DB-WAX and HP-5MS columns. The volatile compounds namely, 3-methyl-1-butanol, 2-methylbutanal, 3-methylbutanal, dimethyl disulfide, 3-(methylthio)-propanal, 3-methylbutanoic acid and benzaldehye were found to be the major contributors to the characteristic odor of *Budu*. Thirty volatiles compounds were detected by both columns, but some compounds detected were different from each other. We can conclude that both columns were suitable for *Budu* analyses since various volatile flavor compounds could be identified. 

The results obtained in this study could provide useful information to predict the quality and safety of commercial *Budu*. This is due to the fact that pH and salt content are generally regarded as the main factors to ensure the safety of food products. In this case, *Budu* that has low pH and high salt concentration is safe for consumption because it may free from pathogenic microbial contaminations. Moreover, some compounds that were harmful for human consumption, such as dimethyl chloride and trichloromethane were detected during volatile compound analyses. Hence, these results can be used to measure the quality and safety level of *Budu*.
